# Heterogeneity in childhood trauma and impulsivity among adolescents with new psychoactive substance use disorders: a latent profile analysis

**DOI:** 10.3389/fpsyg.2026.1918414

**Published:** 2026-08-31

**Authors:** Shumei Zhuang, Huiying Liu, Xiaojuan Che, Kainuo Hou, Yuelin Song, Lehan Li, Yifan Jiang, Shaoqiang Li, Xiaoxu Shang, Changyu Song, Xiaohong Zhang, Jianling Jing, Linjun Shi, Jinyi Fan, Shenshen Qin, Zhiguang Xu, Jianxun Zhang, Jinyuan Li

**Affiliations:** 1 School of Nursing, Tianjin Medical University, Tianjin, China; 2 Tianjin Medical University Cancer Institute & Hospital, National Clinical Research Center for Cancer, Tianjin’s Clinical Research Center for Cancer, Tianjin Medical University, Ministry of Education, Key Laboratory of Cancer Prevention and Therapy, Tianjin, China; 3 Tianjin Drug Administration Bureau, Tianjin, China; 4 Tianjin University of Technology and Education, Tianjin, China; 5 Tianjin Anding Hospital (Tianjin Mental Health Centre), Tianjin, China

**Keywords:** adolescents, childhood trauma, heterogeneity, impulsivity, latent profile analysis, new psychoactive substance use disorders

## Abstract

**Background:**

Adolescent patients with new psychoactive substance use disorders represent a growing public health concern worldwide. Childhood trauma and impulsivity are clinically relevant characteristics among adolescents with new psychoactive substance use disorders, but heterogeneity in their co-occurring patterns remains underexplored.

**Methods:**

A cross-sectional study enrolled 1,026 adolescent patients with new psychoactive substance use disorders from 10 primary community healthcare centers in Tianjin, China. Childhood trauma, impulsivity, and perceived parenting styles were assessed using the CTQ-SF, BIS-11, and s-EMBU-C, respectively. Latent profile analysis was conducted using the item-mean scores of five CTQ-SF and three BIS-11 subscales as eight continuous indicators. Univariable and multivariable R3STEP analyses in Mplus examined factors associated with profile membership while accounting for classification uncertainty.

**Results:**

Three distinct profiles were identified: (1) Low Childhood Trauma–Low Impulsivity (*n* = 645, 62.9%); (2) High Emotional Neglect–High Impulsivity (*n* = 263, 25.6%); and (3) High Childhood Trauma–High Impulsivity (*n* = 118, 11.5%). In the primary multivariable R3STEP model, perceived parenting dimensions showed profile-specific conditional associations with latent profile membership. Associations involving the 12-month clinically documented relapse count were examined in an expanded sensitivity model and treated as exploratory.

**Conclusion:**

This study reveals substantial heterogeneity in childhood trauma and impulsivity among adolescents with new psychoactive substance use disorders. The identification of three distinct profiles provides a preliminary descriptive framework for stratified assessment and hypothesis generation regarding profile-informed interventions. Longitudinal studies are needed to evaluate the stability, clinical utility, and prognostic relevance of these profiles.

## 1 Background

Drug dependence has become a major global public health challenge. Worldwide, an estimated 292 million people use addictive substances (United Nations Office on Drugs and Crime, 2024). Notably, new psychoactive substances (NPS), such as synthetic cannabinoids and stimulants, have rapidly emerged in recent years (Zhuang et al., 2025). Compared with traditional drugs, NPS are associated with higher addiction potential, greater psychoactive potency, and increased risks of severe psychiatric, neurological, and physical harm (Sinha, 2024; Peacock et al., 2019; United Nations Office on Drugs and Crime, 2024). Adolescence represents a critical period of neurodevelopment, during which immature emotional regulation and behavioral control heighten susceptibility to substance use initiation and dependence (Guerin et al., 2020). Consequently, early exposure to NPS during this sensitive period not only risks causing irreversible damage to cognitive function, emotional regulation, and impulse control but also significantly increases the likelihood of adult dependence and a cascade of adverse health, psychological, and social outcomes (Odgers et al., 2008). Furthermore, under the influence of adverse environmental factors such as curiosity and peer pressure, adolescents are particularly prone to impulsive and deviant behaviors, marking this as a critical period for the initiation of high-risk substance use (Degenhardt et al., 2016; Robbins et al., 2024). In China, despite strict legal prohibitions, the incidence of adolescent new psychoactive use disorders is increasing (National Narcotics Control Commission of China, 2024). Given this heightened vulnerability and rising prevalence, identifying modifiable risk factors contributing to NPS addiction in this population is essential.

Childhood abuse (including physical abuse and neglect, emotional abuse and neglect, and sexual abuse) refers to any harm, potential harm, or threat caused to children (Leeb and Fluke, 2015). Evidence suggests that childhood trauma may alter hypothalamic–pituitary–adrenal axis functioning, thereby increasing vulnerability to mental health problems and impaired impulse control (Schär et al., 2022). These adverse effects may, in turn, increase the likelihood of substance use, dependence, suicidal behavior, and other high-risk behaviors, ultimately heightening susceptibility to addiction (Kwok et al., 2019). It is a significant stressor for addiction and can directly or indirectly influence adolescent addictive behaviors (Liu et al., 2025). Approximately one-quarter of children worldwide have experienced at least one form of trauma during their growth (Madigan et al., 2023). Such experiences not only directly impair individual physical and mental health but may also lead to restricted cognitive development, emotional regulation disorders, and various psychological problems (World Health Organization, 2024; Massullo et al., 2023; United Nations Office on Drugs and Crime, 2024), exerting long-term and extensive negative impacts on individuals’ physical and psychological development. Research indicates that traumatic experiences may generate maladaptive defenses against painful events (Gori et al., 2023), prompting individuals to seek pleasure or alleviate psychological distress. These responses may be associated with greater impulsivity and, consequently, increased vulnerability to addiction (Liu et al., 2025).

In addition to childhood adversity, impulsivity is also a powerful risk factor for problem behaviors in adolescents (Fosco et al., 2025). It is not only a core symptom of substance use disorders but also an important susceptibility factor for their onset, development, and maintenance (Johnson et al., 2020; Zhuang et al., 2025). As a multidimensional psychological construct, impulsivity is defined as individuals’ weaker inhibitory control over dominant responses, difficulty in delaying gratification, or engaging in careful thinking at the cognitive, emotional, and behavioral levels (Moeller et al., 2001). Neurobiologically, impulsivity is regulated by neural regions such as the basal ganglia and prefrontal cortex (Guzulaitis and Palmer, 2023), leading to enhanced emotional reactivity, poorer persistence and planning abilities, and a preference for immediate rewards over long-term outcomes (Vassileva and Conrod, 2019). Individuals with higher impulsivity are more prone to abuse and dependence on new drugs, more inclined to pursue immediate rewards (Vassileva and Conrod, 2019). This profile renders individuals more prone to NPS abuse, dependence, and relapse after withdrawal.

This study is grounded in the vulnerability–stress model (Monroe and Simons, 1991), which posits that psychological disorders arise from the interaction between individual vulnerability and external stressors. Within this framework, childhood trauma serves as a potent stressor, while impulsivity represents a key vulnerability trait; childhood trauma and impulsivity may co-occur in distinct configurations among adolescents with NPS-SUD.

However, this interaction does not occur in isolation. According to the ecological systems theory (Bronfenbrenner, 1977), the family is the most direct and influential environment; parenting styles are essential for shaping adolescents’ psychological development (Feng et al., 2021) and constitute key contextual factors for adolescents’ substance use (Sanchez et al., 2026). Parents with an authoritative parenting style provide high levels of emotional support and responsible behavioral guidance, which helps promote good behavior regulation (Dionisi and Dupré, 2023; Sanchez et al., 2026). In contrast, parents with an authoritarian parenting style are extremely strict and have low emotional warmth, which may lead to children’s lack of self-confidence, increase their desire to express themselves, thus exacerbating impulsivity (Schneider and Schenck-Fontaine, 2022), resulting in poor behavioral outcomes (Thompson et al., 2003), and is associated with earlier onset and higher frequency of substance use among adolescents.

Based on the integrated framework of the vulnerability-stress model and ecological systems theory, this study aimed to systematically investigate the formation of risk profiles in adolescents with new psychoactive substance use disorders (NPS-SUD). To move beyond the traditional paradigm that examines childhood trauma and impulsivity as isolated variables, we used a person-centered approach: latent profile analysis (LPA). LPA identifies naturally occurring, heterogeneous subgroups within the population based on individuals’ specific configurations across multiple dimensions of childhood trauma and impulsivity. This approach is crucial for uncovering the clinically meaningful heterogeneity that may underlie divergent clinical phenotypes, illness trajectories, and treatment responses.

**Figure 1:**
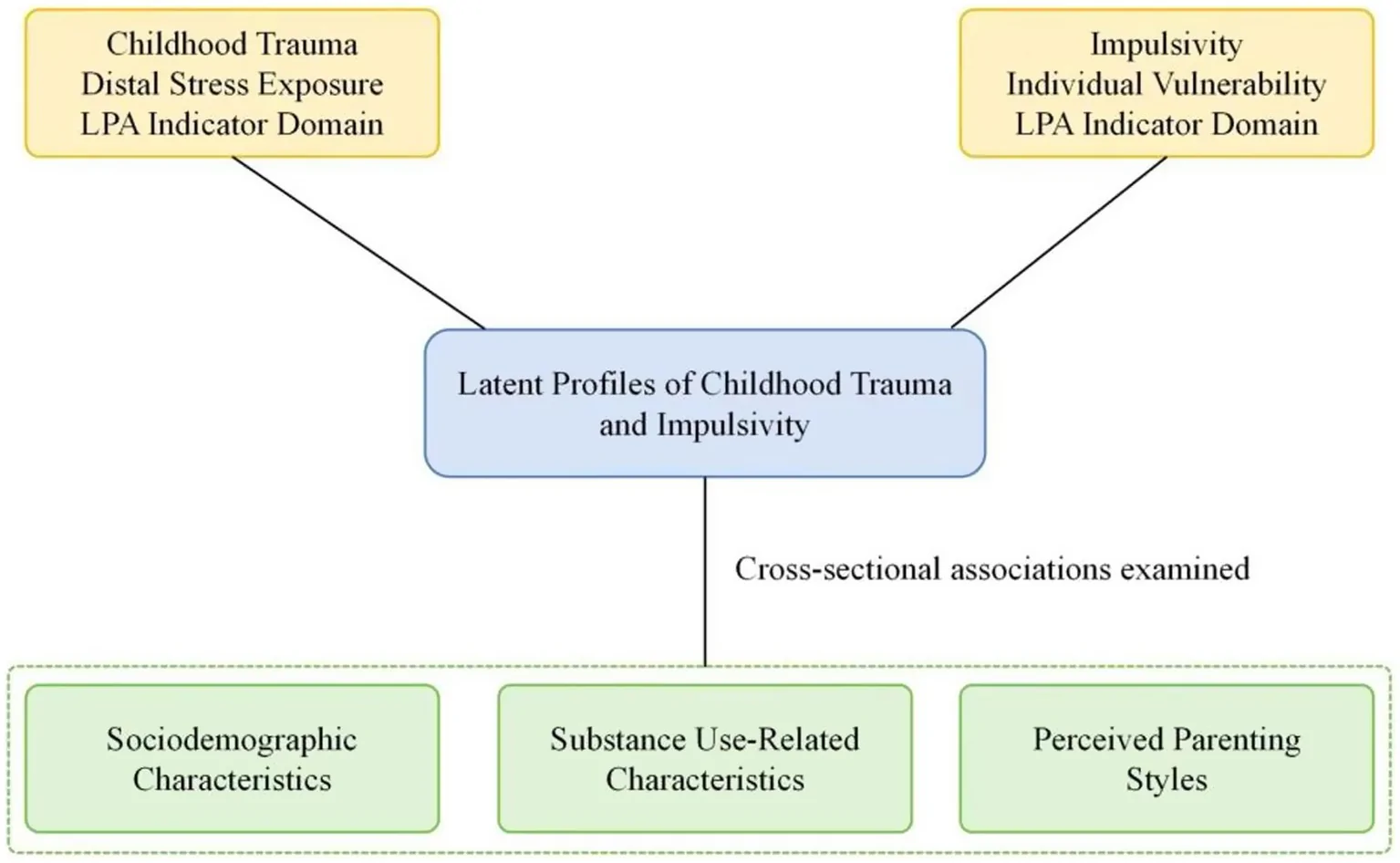
Conceptual framework for latent profile indicators and external correlates.

This study focuses on adolescents with NPS-SUD, a clinically vulnerable population characterized by neurodevelopmental susceptibility, distinct perceptions of NPS-related harm, and potentially complex configurations of early adversity and impulsivity. Grounded in the vulnerability–stress model and ecological systems theory, impulsivity was conceptualized as an individual vulnerability characteristic, childhood trauma as distal stress exposure, and perceived parenting styles as a proximal family-context characteristic. In the present study, childhood trauma and impulsivity were used as indicator domains to identify latent profiles, whereas perceived parenting styles were examined as external correlates of profile membership. This framework was used to organize the study variables and guide hypothesis generation rather than to test causal, moderating, etiological, or prognostic pathways. Accordingly, this study aimed to identify distinct latent profiles of childhood trauma and impulsivity among adolescents with NPS-SUD and to examine the cross-sectional associations of profile membership with sociodemographic characteristics and perceived parenting styles. The association between profile membership and the 12-month clinically documented relapse count was examined as an exploratory analysis. We hypothesized that at least two latent profiles would emerge and that these external characteristics would differ across profiles. The resulting typology was intended to provide a preliminary framework for stratified assessment and profile-informed intervention research (Figure 1).

## 2 Methods

### 2.1 Participants

This multicenter cross-sectional study was conducted from September 2024 to May 2025 across 10 primary community healthcare centers in Tianjin, China. A total of 1,121 questionnaires were distributed to eligible adolescent patients with new psychoactive substance use disorder (NPS-SUD), of which 1,026 were returned and considered valid, yielding a valid response rate of 91.53%. Eligibility was determined based on documented ICD-11 diagnoses established by licensed psychiatrists experienced in the assessment and treatment of substance-related disorders. Routine diagnostic assessment included face-to-face clinical interviews and reviews of patients’ substance-use and medical histories, with available toxicological findings and collateral information considered when clinically indicated. Before enrollment, trained research personnel verified the documented diagnoses and other eligibility information using a common study checklist. The same inclusion and exclusion criteria and eligibility-verification procedures were applied across all participating centers. However, participant-level substance-specific ICD-11 codes were not systematically extracted into the study database; therefore, code-specific distributions could not be reported.

Inclusion criteria were as follows: (1) adolescent participants aged 18–24 years (Sawyer et al., 2018), with age at first NPS use between 10 and 24 years; (2) a clinician-confirmed diagnosis of an NPS-related substance use disorder according to the ICD-11, documented within the previous 12 months; (3) voluntary participation with signed informed consent.

Exclusion criteria were as follows: (1) history of severe craniocerebral injury, neurodegenerative diseases, or other serious physical conditions associated with cognitive impairment; (2) comorbid severe mental illness, such as schizophrenia or bipolar disorder during an acute episode, or a severe neurological disorder; (3) experience of major life events within the past year; (4) participation in other psychological intervention studies within the past 3 months.

### 2.2 Measures

#### 2.2.1 Sociodemographic and NPS use-related characteristics

A researcher-developed questionnaire was used to collect sociodemographic information, including gender, age, educational level, long-term place of residence, monthly per capita household income, and age at first NPS use. Relapse was operationally defined as a clinically documented return to NPS use following a period of abstinence (Stewart, 2008; Substance Abuse and Mental Health Services Administration [SAMHSA], 2023). Relapse information was extracted from participants’ clinical records for the uniform 12-month period immediately preceding the study assessment and was independently verified by two trained researchers. The relapse count represented the actual number of clinically documented relapse episodes occurring during this period.

#### 2.2.2 Childhood Trauma Questionnaire – Short Form (CTQ-SF)

The Childhood Trauma Questionnaire–Short Form (CTQ-SF), developed by Bernstein et al. (2003), is a 28-item self-report instrument used to retrospectively assess childhood traumatic experiences. The scale contains 25 scored items and three minimization–denial validity items. The scored items comprise five subscales, each containing five items: physical abuse, emotional abuse, sexual abuse, physical neglect, and emotional neglect. Items are rated on a 5-point Likert scale ranging from 1 (never true) to 5 (very often true), with higher scores indicating greater exposure to the corresponding form of childhood trauma. The CTQ-SF was initially translated and validated for use in Chinese populations by Zhao et al. (2005), and its psychometric properties were subsequently supported in Chinese clinical and non-clinical samples (He et al., 2019). In the present study, the Cronbach’s *α* coefficient for the CTQ-SF was 0.914. For each subscale, an item-mean score was calculated by averaging the five constituent items. These five item-mean scores were entered into the LPA as continuous indicators without z-score standardization.

#### 2.2.3 Barratt Impulsivity Scale-11 (BIS-11)

The Barratt Impulsiveness Scale–11 (BIS-11) is a 30-item self-report instrument revised by Patton et al. (1995) to assess impulsivity. A Chinese version was adapted and validated by Li et al. (2011). The scale comprises three dimensions: non-planning impulsivity, motor impulsivity, and attentional impulsivity. All items are scored on a 5-point Likert scale (1 = rarely/never, 5 = almost always/always), with higher scores indicating higher levels of impulsivity. The Chinese version has demonstrated good reliability and validity among Chinese populations (Huang et al., 2013). The Cronbach’s *α* coefficient for the BIS-11 in the present study was 0.918. For each subscale, an item-mean score was calculated by dividing the raw subscale sum by the number of constituent items. These three subscale item-mean scores were used as LPA indicators without *z*-score standardization.

#### 2.2.4 Short-form Egna Minnen av Barndoms Uppfostran for Chinese (s-EMBU-C)

The Short-form Egna Minnen av Barndoms Uppfostran for Chinese (s-EMBU-C) is a condensed version of the original Parental Rearing Style Questionnaire (EMBU) developed by Perris et al. (1980), and it was culturally adapted and validated for Chinese samples by Jiang et al. (2010). The scale contains 42 items, equally divided into father and mother versions (21 items each). Both versions include three subscales: rejection, overprotection, and emotional warmth. Items are rated on a 4-point Likert scale (1 = never, 4 = always), with higher scores indicating a higher frequency of the corresponding parenting style. In the present study, the Cronbach’s α coefficients were 0.867 for paternal rejection, 0.835 for paternal emotional warmth, 0.807 for paternal overprotection, 0.834 for maternal rejection, 0.861 for maternal emotional warmth, and 0.832 for maternal overprotection. For the R3STEP analyses, corresponding paternal and maternal subscale total scores were averaged to create composite measures of parental rejection, emotional warmth, and overprotection, thereby reducing redundancy and improving model parsimony.

### 2.3 Ethical approval

Before enrollment, all eligible participants received oral and written information regarding the study purpose, procedures, potential risks and benefits, and data-handling and protection procedures. Participants were given sufficient opportunity to ask questions, and written informed consent was obtained before data collection. Participation was entirely voluntary, and participants could decline participation or withdraw from the study at any time without penalty or any effect on the healthcare, follow-up, or other services to which they were entitled. Psychological support or referral was available when needed. Strict confidentiality procedures were implemented. Electronic data were stored in an encrypted, password-protected database accessible only to authorized research personnel, and all personally identifiable information was removed before data analysis. This study was conducted in accordance with the Declaration of Helsinki, registered at the Chinese Clinical Trial Registry (ChiCTR2400082705), and approved by the ethics committee (TMUhMEC2021013), ensuring compliance with privacy regulations.

### 2.4 Data analysis

The LPA was performed using Mplus version 8.3. For each of the five CTQ-SF subscales and three BIS-11 subscales, an item-mean score was calculated by dividing the subscale sum by the number of constituent items. These eight item-mean scores were entered as continuous indicators without z-score standardization or further transformation. Models with one to five profiles were estimated using robust maximum likelihood estimation. To reduce the risk of local maxima, 200 random sets of starting values were used, with the best 50 retained for final-stage optimization; the best log-likelihood was replicated 10 times. Within-profile indicator variances were constrained to equality across profiles, and within-profile covariances were fixed at zero.

Model selection was based on the Akaike information criterion (AIC), Bayesian information criterion (BIC), sample-size-adjusted BIC (aBIC), entropy, Lo–Mendell–Rubin adjusted likelihood ratio test (LMRT), bootstrap likelihood ratio test (BLRT), profile size, substantive interpretability, and parsimony. Profiles comprising less than 5% of the sample were interpreted cautiously because very small profiles may be unstable or potentially spurious (Ferguson et al., 2020). Lower AIC, BIC, and aBIC values indicated better fit; entropy values closer to 1 indicated greater classification precision; and significant LMRT and BLRT results favored a model with k profiles over the corresponding k-1 profile model. Indicator patterns in the selected solution were described using model-estimated profile-specific means on the original 1–5 item-response scales. Because no formal mixture-model tests of between-profile differences in indicator means were conducted, these comparisons were interpreted descriptively.

Descriptive analyses were conducted using SPSS version 27.0. Categorical variables were summarized as frequencies and percentages, and continuous variables as means and standard deviations. The grouped relapse categories presented in Table 1 were used only to summarize the sample distribution. Associations between candidate covariates and latent profile membership were examined using univariable and multivariable R3STEP analyses in Mplus version 8.3. R3STEP preserved the selected latent profile solution while accounting for classification uncertainty. Univariable models were estimated separately for each candidate covariate; however, statistical significance in the univariable analyses was not used as the sole criterion for multivariable model construction.

**Table 1:** Sociodemographic and NPS use-related characteristics of the participants (*n* = 1,026).

Characteristics	Variables	Patient (*n*%)
Gender	Male	403 (39.28%)
	Female	623 (60.72%)
Age	18–20	453 (44.15%)
	21–24	573 (55.85%)
Education Level	Primary school or below	481 (46.88%)
	Junior middle school	339 (33.04%)
	High school or above	206 (20.08%)
Long-term residence	City	651 (63.45%)
	Town	272 (26.51%)
	Countryside	103 (10.04%)
Monthly per capita household income (CNY)	< 2000	280 (27.29%)
	2001–5,000	274 (26.71%)
	5,001–8,000	247 (24.07%)
	>8,000	225 (21.93%)
Age at first use	10–14	135 (13.16%)
	15–19	286 (27.87%)
	20–24	605 (58.97%)
Number of relapses	0	161 (15.69%)
	1–2	582 (56.72%)
	3–4	215 (20.96%)
	> 4	68 (6.63%)

Relapse was defined as a clinically documented return to NPS use following a period of abstinence. Relapse episodes were identified from clinical records during the uniform 12-month period immediately preceding the study assessment. The categories presented in this table were used solely for descriptive purposes. In the inferential analyses, the actual number of relapse episodes was entered as a continuous covariate in the univariable and expanded sensitivity R3STEP models but was not included in the primary multivariable model.

For the multivariable analyses, parental rejection, parental emotional warmth, and parental overprotection were retained because of their theoretical relevance as family-context characteristics. A parsimonious primary multivariable R3STEP model included these three composite parenting dimensions as continuous covariates. To assess the sensitivity of the primary estimates to recent relapse history, an expanded sensitivity model additionally included the actual number of clinically documented relapse episodes during the 12 months preceding the study assessment. The expanded model was treated as a sensitivity analysis rather than as the primary inferential model.

Pearson correlations and variance inflation factors were examined among the three parenting covariates to assess multicollinearity, and Mahalanobis distance was used to screen for multivariate outliers in the primary model. Model termination, estimation warnings, extreme coefficients, non-estimable standard errors, unusually wide confidence intervals, and restricted covariate distributions were examined as potential indications of sparse-data instability or possible separation. As a scaling sensitivity analysis, the continuous covariates were also standardized and the models were re-estimated.

The Low Childhood Trauma-Low Impulsivity profile served as the reference category in comparisons with the High Emotional Neglect–High Impulsivity and High Childhood Trauma-High Impulsivity profiles; the latter two profiles were also compared directly. Results are reported as unstandardized regression coefficients, standard errors, odds ratios, and 95% confidence intervals. For the parenting variables, the OR represented the change in the odds of profile membership associated with a one-point increase in the corresponding composite score, whereas the OR for relapse represented the change associated with one additional clinically documented relapse episode. All tests were two-sided, with *p* < 0.05 indicating statistical significance.

## 3 Results

### 3.1 Sample characteristics

A total of 1,026 participants were included in this study, with a mean age of 20.97 ± 2.00 years and a mean age at first NPS use of 19.52 ± 3.84 years. There were 403 males (39.28%) and 623 females (60.72%). Additional sociodemographic characteristics are presented in Table 1.

### 3.2 Latent profile enumeration and profile characterization

Based on the fit indices, the AIC, BIC, and aBIC values decreased progressively as the number of profiles increased from one to five. The LMRT and BLRT also remained statistically significant for the four- and five-profile solutions, indicating improved statistical fit relative to models with fewer profiles (Table 2). Therefore, the three-profile solution was not selected solely on the basis of statistical fit indices. Instead, the final solution was determined through joint consideration of statistical fit, classification quality, profile size, substantive distinctiveness, theoretical interpretability, and parsimony.

**Table 2:** Fit indices for the one- to five-profile solutions.

Number of profiles	AIC	BIC	aBIC	Entropy	LMRT(p)	BLRT(p)	Profile proportions	LIP (%)	LIPpp
1	21291.019	21369.954	21319.136	–	–	–	–	–	–
2	19334.981	19458.317	19378.914	0.993	<0.001	<0.001	0.882, 0.118	9.286	1.032
3	18564.137	18731.873	18623.885	0.917	<0.001	<0.001	0.629, 0.256, 0.115	4.090	0.454
4	17712.151	17924.289	17787.716	0.974	0.002	<0.001	0.767, 0.060, 0.068, 0.104	4.704	0.523
5	17186.405	17442.943	17277.786	0.941	0.009	<0.001	0.613, 0.054, 0.209, 0.080, 0.045	3.085	0.343

AIC = Akaike Information Criterion; BIC = Bayesian Information Criterion; aBIC = adjusted Bayesian Information Criterion; LMRT (p) = *p*-value for the Lo–Mendell–Rubin; BLRT (p) = *p*-value for the Bootstrapped Likelihood Ratio Test; LIP = likelihood increment percentage; LIPpp = likelihood increment percentage per additional parameter. LIPpp was calculated by dividing the LIP by the number of additional free parameters relative to the preceding model.

The three-profile solution had an entropy of 0.917 and identified three profiles comprising 645, 263, and 118 participants, representing 62.9, 25.6, and 11.5% of the sample, respectively. The average posterior probabilities were 0.967, 0.942, and 0.987, indicating satisfactory classification precision (Table 3). The three profiles also showed clearly distinguishable and theoretically coherent patterns across the childhood trauma and impulsivity indicators.

**Table 3:** Average posterior probabilities for latent profile membership.

Assigned profile	Class 1	Class 2	Class 3
Class 1	0.967	0.032	0.000
Class 2	0.052	0.942	0.006
Class 3	0.000	0.012	0.987

Class 1 = Low Childhood Trauma–Low Impulsivity profile; Class 2 = High Emotional Neglect–High Impulsivity profile; Class 3 = High Childhood Trauma–High Impulsivity profile.

The four-profile solution had an entropy of 0.974 and included profiles representing 76.7, 6.0, 6.8, and 10.4% of the sample. Although the transition from three to four profiles yielded LIP and LIPpp values of 4.704% and 0.523, respectively, examination of the profile patterns indicated that the additional profiles mainly represented further subdivisions of the elevated childhood trauma and impulsivity configurations identified in the three-profile solution. Several indicator trajectories showed substantial overlap and crossing, and the additional profiles were less clearly differentiated and more difficult to interpret independently.

The five-profile solution had an entropy of 0.941 and yielded profile proportions of 61.3, 5.4, 20.9, 8.0, and 4.5%. Previous methodological guidance has suggested that profiles comprising less than 5% of the sample may be unstable or potentially spurious and should therefore be interpreted with caution (Ferguson et al., 2020). Accordingly, the 4.5% profile raised concern regarding possible overextraction, although profile size alone was not considered sufficient to reject the solution. Moreover, the LIP and LIPpp values decreased to 3.085% and 0.343, respectively, indicating a smaller incremental improvement than that obtained by adding the fourth profile. Examination of the five-profile patterns further showed that the additional profiles primarily subdivided existing trauma–impulsivity configurations and introduced greater overlap without yielding clearly distinct patterns with independent theoretical or clinical meaning.

The four- and five-profile solutions were therefore not rejected because of numerical non-convergence or statistical instability. Rather, considering statistical adequacy, classification precision, profile size, substantive distinctiveness, theoretical interpretability, and parsimony together, the three-profile solution was retained as the most appropriate representation of the data.

Based on the model-estimated indicator patterns, the three profiles were labeled as follows: Class 1, Low Childhood Trauma–Low Impulsivity (*n* = 645, 62.9%); Class 2, High Emotional Neglect–High Impulsivity (*n* = 263, 25.6%); and Class 3, High Childhood Trauma–High Impulsivity (*n* = 118, 11.5%).

**Figure 2:**
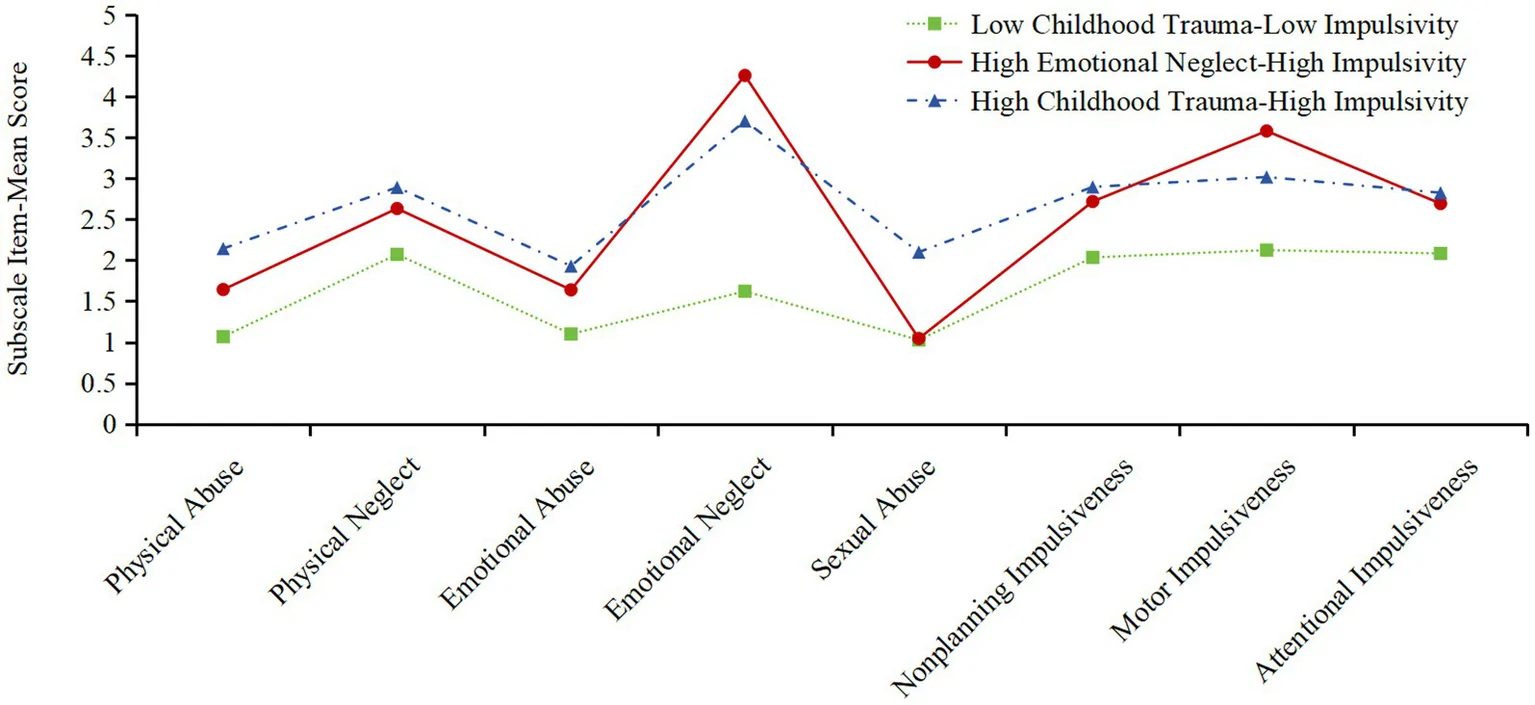
Latent profiles of childhood trauma and impulsivity. The *x*-axis represents the five CTQ-SF subscales and the three BIS-11 subscales. Subscale item-mean scores were calculated by averaging the items within each subscale. The plotted values represent the model-estimated profile-specific means of the eight indicators. No z-score standardization was performed.

Figure 2 presents the model-estimated means of the eight item-mean indicators across the three latent profiles. The indicators are shown on their original item-response scales and were not *z*-score standardized. The High Childhood Trauma-High Impulsivity profile showed comparatively higher levels of emotional abuse, physical neglect, and attentional impulsivity than the other two profiles, indicating a pattern of broader childhood trauma accompanied by elevated attentional impulsivity. The High Emotional Neglect–High Impulsivity profile was characterized by relatively higher scores on emotional neglect and motor impulsivity, indicating the co-occurrence of emotional deprivation and behavioral impulsivity. In contrast, the Low Childhood Trauma–Low Impulsivity profile showed the lowest scores across all eight indicators, representing comparatively lower levels of childhood trauma and impulsivity within this clinical sample. These descriptive patterns illustrate the distinct configurations of childhood trauma and impulsivity across the three latent profiles.

### 3.3 Univariable R3STEP analyses of external characteristics associated with latent profile membership

Univariable R3STEP analyses indicated that gender, selected categories of educational level, place of residence, monthly per capita household income, the 12-month clinically documented relapse count, and the three composite parenting dimensions were associated with latent profile membership in at least one pairwise comparison. Neither current age nor age at first NPS use was significantly associated with profile membership in any pairwise comparison.

Females had higher odds of membership in Class 2 than in Class 1 but lower odds of membership in Class 3 than in Class 2. A high-school education or above was associated with lower odds of Class 3 membership relative to Classes 1 and 2, while town residence was associated with higher odds of Class 2 membership relative to Class 1. Compared with an income of <CNY 2,000, monthly per capita household incomes of CNY 2,001–5,000 and >CNY 8,000 were associated with profile membership in selected comparisons. The 12-month clinically documented relapse count was inversely associated with Class 2 versus Class 1 membership but positively associated with Class 3 versus Class 2 membership.

Higher parental rejection was associated with greater odds of membership in Classes 2 and 3 relative to Class 1. Higher parental emotional warmth was consistently associated with lower odds across all three pairwise comparisons, whereas higher parental overprotection was associated with lower odds of membership in Classes 2 and 3 relative to Class 1. Detailed estimates are presented in Table 4.

**Table 4:** Univariable R3STEP analyses of characteristics associated with latent profile membership.

Variable	Class 2 vs. Class 1		Class 3 vs. Class 1		Class 3 vs. Class 2	
	OR (95% CI)	*p*	OR (95% CI)	*p*	OR (95% CI)	*p*
Gender: Female vs. male (ref.)	1.666 (1.187–2.337)	0.003	0.697 (0.467–1.039)	0.076	0.418 (0.262–0.667)	<0.001
Age, per 1-year increase	1.044 (0.964–1.132)	0.291	1.047 (0.950–1.155)	0.355	1.003 (0.896–1.122)	0.961
Education level (ref.: Primary school or below)						
Junior middle school	0.876 (0.609–1.259)	0.474	0.945 (0.613–1.458)	0.799	1.079 (0.656–1.775)	0.764
High school or above	0.988 (0.656–1.487)	0.954	0.447 (0.234–0.853)	0.015	0.452 (0.224–0.912)	0.027
Place of residence (ref.: City)						
Town	1.569 (1.098–2.241)	0.013	1.461 (0.940–2.271)	0.092	0.931 (0.571–1.521)	0.777
Countryside	1.010 (0.592–1.722)	0.971	0.477 (0.196–1.162)	0.103	0.472 (0.179–1.243)	0.129
Monthly per capita household income, CNY (ref.: <2000)						
2001–5,000	3.058 (1.986–4.707)	<0.001	5.718 (3.029–10.796)	<0.001	1.870 (0.941–3.717)	0.074
5,001–8,000	0.755 (0.460–1.240)	0.267	1.569 (0.776–3.175)	0.210	2.078 (0.925–4.667)	0.076
>8,000	1.614 (1.017–2.563)	0.042	3.509 (1.810–6.801)	<0.001	2.173 (1.040–4.540)	0.039
Age at first NPS use, per 1-year increase	1.008 (0.968–1.049)	0.713	1.037 (0.983–1.095)	0.184	1.029 (0.969–1.093)	0.344
Number of relapses, per one-relapse increase	0.798 (0.673–0.947)	0.010	0.983 (0.876–1.104)	0.772	1.231 (1.014–1.495)	0.035
Perceived Parenting Dimensions						
Parental rejection	1.280 (1.206–1.359)	<0.001	1.173 (1.083–1.269)	<0.001	0.916 (0.834–1.007)	0.068
Parental emotional warmth	0.358 (0.302–0.424)	<0.001	0.105 (0.066–0.168)	<0.001	0.294 (0.192–0.449)	<0.001
Parental overprotection	0.734 (0.686–0.784)	<0.001	0.737 (0.704–0.770)	<0.001	1.004 (0.940–1.073)	0.902

Each characteristic was examined separately using the R3STEP procedure, which accounts for uncertainty in latent profile classification. Class 1 = Low Childhood Trauma–Low Impulsivity profile; Class 2 = High Emotional Neglect–High Impulsivity profile; Class 3 = High Childhood Trauma–High Impulsivity profile. Parental rejection, emotional warmth, and overprotection are composite scores derived from the corresponding paternal and maternal subscales. OR = odds ratio; CI = confidence interval; ref. = reference category; CNY = Chinese yuan.

### 3.4 Multivariable R3STEP analysis and sensitivity analysis

The absolute Pearson correlations among the three composite parenting dimensions ranged from 0.280 to 0.445, and the variance inflation factors ranged from 1.021 to 1.877. In the primary three-predictor model, the maximum Mahalanobis distance was 16.117, which was below the critical value of *χ^2^*(3) = 16.266 at *p* < 0.001. All coefficients and standard errors were estimable, and no unusually wide confidence intervals were observed. Overall, these diagnostic results did not indicate problematic multicollinearity or obvious multivariate outliers in the primary model.

The primary multivariable R3STEP model included parental rejection, parental emotional warmth, and parental overprotection (Table 5). Compared with Class 1, membership in Class 2 was conditionally associated with lower parental emotional warmth (OR = 0.370, 95% CI: 0.294–0.466, *p* < 0.001). Parental rejection (OR = 1.069, 95% CI: 0.786–1.454, *p* = 0.670) and parental overprotection (OR = 1.048, 95% CI: 0.781–1.408, *p* = 0.753) were not statistically significant.

**Table 5:** Primary multivariable R3STEP analysis of factors conditionally associated with latent profile membership.

Profile comparison	Variable	*B*	SE	*z*	*p*	OR	95% CI
Class 2 vs. Class 1	Parental rejection	0.067	0.157	0.426	0.670	1.069	0.786–1.454
	Parental emotional warmth	−0.994	0.118	−8.447	< 0.001	0.370	0.294–0.466
	Parental overprotection	0.047	0.150	0.315	0.753	1.048	0.781–1.408
Class 3 vs. Class 1	Parental rejection	−0.279	0.165	−1.691	0.091	0.757	0.547–1.045
	Parental emotional warmth	−2.799	0.236	−11.874	< 0.001	0.061	0.038–0.097
	Parental overprotection	0.680	0.165	4.109	< 0.001	1.974	1.427–2.730
Class 3 vs. Class 2	Parental rejection	−0.346	0.053	−6.501	< 0.001	0.708	0.638–0.785
	Parental emotional warmth	−1.805	0.202	−8.947	< 0.001	0.165	0.111–0.244
	Parental overprotection	0.633	0.069	9.107	< 0.001	1.882	1.643–2.157

Class 1 = Low Childhood Trauma-Low Impulsivity profile; Class 2 = High Emotional Neglect-High Impulsivity profile; Class 3 = High Childhood Trauma-High Impulsivity profile. The R3STEP procedure accounts for uncertainty in latent profile classification. Parental rejection, emotional warmth, and overprotection are composite scores derived by averaging the corresponding paternal and maternal subscale total scores. B = unstandardized regression coefficient; SE = standard error; z = B/SE; OR = odds ratio; CI = confidence interval.

Compared with Class 1, membership in Class 3 was conditionally associated with lower parental emotional warmth (OR = 0.061, 95% CI: 0.038–0.097, *p* < 0.001) and higher parental overprotection (OR = 1.974, 95% CI: 1.427–2.730, *p* < 0.001). Parental rejection was not statistically significant in this comparison (OR = 0.757, 95% CI: 0.547–1.045, *p* = 0.091).

Compared with Class 2, membership in Class 3 was conditionally associated with lower parental rejection (OR = 0.708, 95% CI: 0.638–0.785, *p* < 0.001), lower parental emotional warmth (OR = 0.165, 95% CI: 0.111–0.244, *p* < 0.001), and higher parental overprotection (OR = 1.882, 95% CI: 1.643–2.157, *p* < 0.001).

An expanded sensitivity model additionally included the actual number of clinically documented relapse episodes during the 12 months preceding the study assessment (Supplementary Table S1). Each additional relapse episode was associated with lower odds of membership in Class 2 versus Class 1 (OR = 0.212, 95% CI: 0.078–0.578, *p* = 0.002), Class 3 versus Class 1 (OR = 0.096, 95% CI: 0.032–0.290, *p* < 0.001), and Class 3 versus Class 2 (OR = 0.454, 95% CI: 0.287–0.719, *p* = 0.001).

However, adding the relapse count substantially changed the magnitude and statistical significance of several parenting-related estimates. Most notably, the OR for parental emotional warmth in the Class 3 versus Class 1 comparison decreased from 0.061 in the primary model to 0.006 in the expanded model. The estimate for parental overprotection in the Class 2 versus Class 1 comparison also changed direction. Standardization of the continuous covariates did not eliminate these extreme estimates, suggesting that differences in variable scaling were not the sole explanation. The expanded model was therefore interpreted as exploratory rather than as the principal basis for the study conclusions.

## 4 Discussion

The present study identified three distinct latent profiles of childhood trauma and impulsivity among adolescents with NPS-SUD. In the primary classification-error-adjusted analysis, perceived parenting dimensions showed profile-specific conditional associations with latent profile membership. The 12-month clinically documented relapse count showed additional associations in the expanded sensitivity analysis; however, the corresponding estimates were sensitive to model specification and were therefore regarded as exploratory.

The three latent profiles represented distinct configurations of childhood trauma and impulsivity. The Low Childhood Trauma-Low Impulsivity profile, which comprised the majority of the sample, showed comparatively low levels across the five childhood trauma dimensions and the three impulsivity dimensions, representing a comparatively lower trauma-impulsivity configuration within this clinical sample. The High Emotional Neglect-High Impulsivity profile was characterized primarily by emotional neglect and motor impulsivity, reflecting the co-occurrence of emotional deprivation and behavioral disinhibition. Adolescent neglect has been associated with subsequent substance use through impaired self-regulation and a stronger preference for immediate rewards, while psychological abuse and neglect are interconnected with multiple dimensions of impulsivity (Peviani et al., 2024; Wu et al., 2025). The High Childhood Trauma-High Impulsivity profile showed broadly elevated levels across multiple childhood trauma and impulsivity dimensions, indicating the co-occurrence of pervasive early adversity and pronounced self-regulatory difficulties. Childhood maltreatment is associated with an increased risk of subsequent substance use and misuse among young people (Rakovski et al., 2024), and impulsivity may represent an important psychological pathway linking childhood maltreatment to addictive behaviors in adolescents (Liu et al., 2025).

Perceived parenting dimensions showed profile-specific conditional associations in the primary multivariable R3STEP model. Compared with the Low Childhood Trauma-Low Impulsivity profile, the High Emotional Neglect-High Impulsivity profile was associated with lower parental emotional warmth, whereas parental rejection and overprotection were not statistically significant. This pattern highlights the potential relevance of insufficient emotional support, as greater parental warmth has been associated with lower levels of substance use among adolescents and emerging adults (Pinquart and Gochaliyev, 2026). Neuroimaging evidence further suggests that parental acceptance and warmth are associated with patterns of default mode network connectivity linked to fewer externalizing symptoms in youth (Davis et al., 2022).

Compared with the Low Childhood Trauma-Low Impulsivity profile, the High Childhood Trauma-High Impulsivity profile was associated with lower parental emotional warmth and higher parental overprotection, whereas parental rejection was not statistically significant. The combination of lower parental emotional warmth and higher overprotection may reflect a family context perceived as providing limited emotional support while constraining adolescents’ autonomy. Overprotective parenting has been modestly associated with early maladaptive schemas involving impaired autonomy and performance and disconnection and rejection, particularly enmeshment, emotional deprivation, and mistrust or abuse (Bruysters and Pilkington, 2023).

Compared with the High Emotional Neglect-High Impulsivity profile, the High Childhood Trauma-High Impulsivity profile was characterized by lower parental rejection and emotional warmth but higher parental overprotection. Although the inverse association with parental rejection may appear inconsistent with the descriptive profile pattern, it represents a conditional association estimated after adjustment for the other parenting dimensions and may reflect shared variance or suppression among the predictors. Previous evidence nevertheless indicates that maternal and paternal rejection are associated with greater internalizing and externalizing problems among children and adolescents (Rothenberg et al., 2022).

Taken together, these findings suggest that emotional warmth, rejection, and overprotection may be better understood as an interrelated pattern of perceived parenting rather than as isolated dimensions. These findings should nevertheless be interpreted at the overall parenting level, because the corresponding paternal and maternal subscale total scores were averaged and parent-specific associations were not examined.

In the expanded R3STEP sensitivity analysis, the 12-month clinically documented relapse count was additionally examined alongside the three perceived parenting dimensions. Each additional documented relapse episode was associated with lower odds in all three pairwise profile comparisons. However, including relapse materially altered the magnitude and statistical significance of several parenting-related estimates, indicating sensitivity to model specification. Accordingly, the relapse-related estimates were treated as exploratory. Although recent relapse history may be associated with profile membership, the cross-sectional design and model sensitivity preclude conclusions regarding temporal or causal relationships. Moreover, despite the consistent operational definition and uniform 12-month observation period, the documented relapse count alone may not fully capture clinically relevant features such as episode duration, severity, substance type, treatment exposure, or relapse context (Maisto et al., 2003; Chung and Maisto, 2006).

### 4.1 Clinical implications

The three latent profiles identified in this study may provide a preliminary framework for stratified assessment and hypothesis generation regarding intervention planning among adolescents with NPS-SUD. Clinical assessment may consider the combined patterns of childhood trauma and impulsivity rather than relying exclusively on total scores or isolated indicators. However, profile membership should complement, rather than replace, individualized clinical evaluation.

For individuals in the Low Childhood Trauma-Low Impulsivity profile, relatively lower levels of trauma and impulsivity should not be assumed to indicate limited clinical needs. Assessment may therefore extend to current substance-use patterns, recovery capital, social-network composition, previous treatment experiences, and continuing-care needs. Recovery capital and social-network characteristics are relevant to treatment engagement and post-treatment transitions among adolescents receiving substance-use treatment, while the broader heterogeneity of SUD supports a multidimensional rather than single-factor assessment approach (Hennessy et al., 2026; Guerrin et al., 2025). Continuing-care strategies, including post-treatment follow-up, reassessment, and linkage to additional services when needed, may also be incorporated into relapse-prevention planning (Gaolaolwe et al., 2025).

For individuals in the High Emotional Neglect-High Impulsivity profile, assessment and intervention may place greater emphasis on perceived emotional support, family functioning, and impulse-control difficulties. Higher perceived social support during adolescence may attenuate the association between adverse childhood experiences and subsequent problematic alcohol and drug use (Rogers et al., 2023). Ecological family-based treatments and individual or group cognitive-behavioral therapies may therefore represent relevant outpatient treatment options for adolescents with substance-use problems (Hogue et al., 2026).

For individuals in the High Childhood Trauma-High Impulsivity profile, trauma-informed interventions that address both trauma-related symptoms and substance use, with family involvement when appropriate, may be particularly relevant. Risk Reduction through Family Therapy integrates trauma-focused, substance-use, and family-based components and has demonstrated reductions in substance-use days and selected posttraumatic stress symptom domains among trauma-exposed adolescents (Danielson et al., 2020). Trauma-focused interventions may also reduce externalizing behavioral problems among adolescents with traumatic experiences (Jiang et al., 2025).

These profile-informed considerations should be regarded as preliminary and hypothesis-generating. Their clinical effectiveness should be evaluated prospectively, and the profiles should not be used as stand-alone criteria for treatment allocation.

### 4.2 Limitations

This study has several limitations. First, the cross-sectional design precludes conclusions regarding causality, temporal relationships, or prognosis. Second, the reliance on self-administered questionnaires may have introduced recall and reporting bias. Third, the exclusion of patients with severe psychiatric or neurological comorbidities may limit the applicability of the findings to individuals with more complex clinical conditions. Fourth, participant-level data on the primary NPS type, polysubstance use, and substance-specific ICD-11 diagnoses and codes were not systematically recorded across centers, precluding reliable determination of their distributions. No study-specific structured diagnostic interview was conducted, and inter-rater reliability was not assessed. Although relapse was defined consistently and assessed from clinical records over a uniform 12-month period, undocumented episodes may have been missed, and relapse severity and context were not captured. Future multicenter studies should prospectively record detailed substance-use characteristics and complete ICD-11 diagnostic information. Fifth, recruitment from 10 primary community healthcare centers in a single region, together with limited ethnic and sociocultural diversity, may restrict generalizability. Because center identifiers were not retained, within-center clustering could not be accounted for. Finally, estimates from the expanded R3STEP sensitivity model were sensitive to covariate specification. Given the relatively small size of Profile 3 and possible sparse-data effects, associations involving the relapse count should be interpreted as exploratory. Future studies should use longitudinal and multicenter designs, prospectively collect more detailed diagnostic, substance-use, treatment, and relapse information from multiple sources, employ structured diagnostic assessments and evaluate inter-rater reliability, retain center identifiers, and validate the present findings in larger and more balanced samples.

## 5 Conclusion

In conclusion, this study identified three empirically derived profiles representing distinct configurations of childhood trauma and impulsivity among adolescents with NPS-SUD. In the primary classification-error-adjusted analysis, perceived parenting dimensions showed profile-specific conditional associations with latent profile membership. The clinically documented relapse count showed additional associations in an expanded sensitivity analysis, although these findings were sensitive to model specification and should be considered exploratory. The identified profiles provide a preliminary descriptive framework for stratified clinical assessment and future intervention research. Longitudinal studies with larger and more balanced samples are needed to examine profile stability, temporal associations, clinical utility, and relationships with subsequent treatment and recovery outcomes while accounting for classification uncertainty.

## Data Availability

The data supporting the findings of this study are not publicly available because they contain sensitive information and are subject to privacy protection regulations. To safeguard participant privacy and comply with ethical review board requirements, data sharing is restricted. Enquiries regarding the data can be directed to the corresponding author/s.
