# Supplementary material for: Heterogeneity in childhood trauma and impulsivity among adolescents with new psychoactive substance use disorders: a latent profile analysis
Source: Front Psychol. 2026 Aug 31;17:1918414. doi: 10.3389/fpsyg.2026.1918414 (PMC13572711; doi:10.3389/fpsyg.2026.1918414)
Supplement: Supplementary file 1 [file Table_1.docx]

**Supplementary Table S1. *Expanded multivariable R3STEP sensitivity analysis additionally including the 12-month clinically documented relapse count***

| ***Profile comparison*** | ***Variable*** | ***B*** | ***SE*** | ***z*** | ***p*** | ***OR*** | ***95% CI*** |
| --- | --- | --- | --- | --- | --- | --- | --- |
| Class 2 vs Class 1 | Parental rejection | 0.260 | 0.133 | 1.955 | 0.051 | 1.297 | 0.999–1.684 |
|  | Parental emotional warmth | −1.471 | 0.288 | −5.103 | <0.001 | 0.230 | 0.131–0.404 |
|  | Parental overprotection | −0.265 | 0.124 | −2.147 | 0.032 | 0.767 | 0.602–0.977 |
|  | Number of relapses | −1.553 | 0.512 | −3.032 | 0.002 | 0.212 | 0.078–0.578 |
| Class 3 vs Class 1 | Parental rejection | −0.668 | 0.227 | −2.946 | 0.003 | 0.513 | 0.329–0.800 |
|  | Parental emotional warmth | −5.079 | 0.656 | −7.742 | <0.001 | 0.006 | 0.002–0.023 |
|  | Parental overprotection | 0.256 | 0.148 | 1.730 | 0.084 | 1.291 | 0.967–1.726 |
|  | Number of relapses | −2.342 | 0.562 | −4.163 | <0.001 | 0.096 | 0.032–0.290 |
| Class 3 vs Class 2 | Parental rejection | −0.928 | 0.185 | −5.031 | <0.001 | 0.395 | 0.275–0.567 |
|  | Parental emotional warmth | −3.608 | 0.589 | −6.127 | <0.001 | 0.027 | 0.009–0.086 |
|  | Parental overprotection | 0.521 | 0.083 | 6.306 | <0.001 | 1.684 | 1.432–1.980 |
|  | Number of relapses | −0.789 | 0.234 | −3.370 | 0.001 | 0.454 | 0.287–0.719 |

***Note*** Class 1 = Low Childhood Trauma-Low Impulsivity profile; Class 2 = High Emotional Neglect-High Impulsivity profile; Class 3 = High Childhood Trauma-High Impulsivity profile. The expanded sensitivity model included parental rejection, emotional warmth, overprotection, and the actual number of clinically documented relapse episodes during the preceding 12 months. Parenting variables were calculated by averaging the corresponding paternal and maternal subscale total scores. B = unstandardized regression coefficient; SE = standard error; OR = odds ratio; CI = confidence interval.
